# A Retrospective Cohort Study of Lesion Distribution of HIV-1 Infection Patients With Cryptococcal Meningoencephalitis on MRI: Correlation With Immunity and Immune Reconstitution

**DOI:** 10.1097/MD.0000000000002654

**Published:** 2016-02-12

**Authors:** Shuang Xia, Xueqin Li, Yanbin Shi, Jinxin Liu, Mengjie Zhang, Tenghui Gu, Shinong Pan, Liucun Song, Jinsheng Xu, Yan Sun, Qingxia Zhao, Zhiyan Lu, Puxuan Lu, Hongjun Li

**Affiliations:** From the Department of Radiology, Tianjin First Central Hospital (SX, MZ, TG), Tianjin; Department of Radiology, Youan Hospital Affiliated of Capital Medical University (XL, HL), Xitoutiao, Youan Menwai, Beijing; Department of Radiology, Zhengzhou Sixth People's Hospital (YS, LS), Zhengzhou, Henan Province; Department of Radiology, Guangzhou Eighth People's Hospital (JL), Guangzhou, Guangdong Province; Department of Radiology, Shengjing Hospital of China Medical University (SP), Shenyang, Liaoning Province; Department of Infection, Zhengzhou Sixth People's Hospital (JX, YS, QZ), Zhengzhou, Henan Province; Department of Radiology, Zhongnan Hospital of Wuhan University (ZL), Wuhan, Hubei Province; and Department of Radiology, Shenzhen Third People's Hospital of Guangdong Medical College (PL), Shenzhen, Guangdong Province.

## Abstract

The objective of this paper is to correlate the MRI distribution of cryptococcal meningoencephalitis in HIV-1 infection patients with CD4 T cell count and immune reconstitution effect.

A large retrospective cohort study of HIV patients from multi-HIV centers in China was studied to demonstrate the MRI distribution of cryptococcal meningoencephalitis and its correlation with the different immune status.

The consecutive clinical and neuroimaging data of 55 HIV-1-infected patients with cryptococcal meningoencephalitis collected at multi-HIV centers in China during the years of 2011 to 2014 was retrospectively analyzed. The enrolled patients were divided into 2 groups based on the distribution of lesions. One group of patients had their lesions at the central brain (group 1, n = 34) and the other group of patients had their lesions at the superficial brain (group 2, n = 21). We explored their MRI characterization of brain. In addition, we also compared their CD4 T cell counts and immune reconstitution effects between the 2 groups based on the imaging findings.

No statistical difference was found in terms of age and gender between the 2 groups. The medians of CD4 T cell counts were 11.67 cells/mm^3^ (3.00–52.00 cells/mm^3^) in group 1 and 42.00 cells/mm^3^ (10.00–252.00 cells/mm^3^) in group 2. Statistical difference of CD4 T cell count was found between the 2 groups (*P* = 0.023). Thirteen patients in group 1 (13/34) and 12 patients in group 2 (12/21) received highly active antiretroviral treatment (HAART). Patients of group 2 received HAART therapy more frequently than patients of group 1 (*P* = 0.021).

Central and superficial brain lesions detected by MR imaging in HIV-1-infected patients with cryptococcal meningoencephalitis are in correlation with the host immunity and HAART therapy.

## INTRODUCTION

*Cryptococcus neoforms* (*C. neoformans*) infection is a common invasive fungal disease in immunodeficiency patients. Currently, it affects >1 million persons per year and ∼6,50,000 deaths in sub-Saharan Africa.^1,2^ Cryptococcal meningoencephalitis occurs in 40% to 68% of HIV-infected patients and is the most common fungal infection complicating HIV/AIDS.^3–6^ Although the mortality rate is declining along with the use of HAART, many patients die from the development of brain lesions.^7–10^ Therefore, the early detection of cerebral involvement of cryptococcal infection is critically important for directing treatment and predicting the prognosis. Although fungal infection of central nervous system (CNS) rarely occurs in immunocompetent patients, its incidence is increasing in patients with AIDS. Among various fungal infections, cryptococcal meningoencephalitis is most common in patients with HIV/AIDS, whose pathogenic organism is commonly found in the excrements of birds and invades the human CNS via inhalation and hematogenous spread.

The typical MRI signs of CNS cryptococcosis complicating AIDS before the use of HAART are dilated Virchow–Robin spaces and cryptococcomas.^11–13^ In these reports, soap-bubble such as lesions were described in the basal ganglia, midbrain peduncles, and nucleus dentate, indicating clusters of gelatinous pseudocysts.^14^ However, HAART is associated with decreased incidence of opportunistic infection, and in those that develop CNS Cryptococcus there may be a different manifestation in patients based on their HAART treatment.

Limited reports so far focus on radiological findings of cerebral cryptococcosis in HIV-infected patients during the treatment by HAART.^13,15,16^ In one of these reports, a case with cryptococcal meningoencephalitis is described, with leptomeningeal enhancement and parenchymal edema by MR imaging.^17^ In another report, the imaging findings in a larger cohort of 62 patients are analyzed for initial severity of the disease. It is found that high serum and/or CSF antigen titers are correlated with abnormalities demonstrated by MRI and HAART treatment fails to correlate with abnormalities demonstrated by MR imaging due to insufficiency of MRI data.^18^ It has also been reported that leptomeningitis or meningoencephalitis are detected in all patients receiving HAART.^19^ Other imaging findings include multiple cerebral cryptococcomas associated with immune reconstitution.^20^ However, the previous reports either are based on limited sample size or are single case report. More data is needed to compare the HAART treatment effect on imaging characterization of CNS cryptococcal infection.

CD4 T cells play an important role in the occurrence of opportunistic infections in HIV infected patients and the risk of opportunistic infection is especially high when CD4 T cell count is <100 cells/mm^3^.^21^ The growth of *C. neoformans* in the CNS has been found to be inhibited when CD4 T cells are present.^22,23^ However, no evidence has been reported showing the effect of CD4 T cell count on the imaging of crytococcal infection.

We hypothesized that the treatment of HAART and CD4 T cell count might affect the imaging characterization, especially lesion distribution of CNS cryptococcal infection in patients with AIDS. Based on the hypothesis and limitations of previous reports, we collected MRI data of CNS cryptococcal infection from a large cohort of patients that were enrolled from the hospitals specialized in infectious diseases in different regions of China. We also described the lesion distribution and compared the MRI characterization with the clinical data, such as CD4 T cell count and immune reconstitution effect.

## MATERIALS AND METHODS

A total of 75 patients with AIDS and initial episodes of CNS cryptococcal meningoencephalitis between September 2012 and October 2014 were recruited into the study. All of the data were from different local hospitals specialized in infectious diseases in China and finally were analyzed at Department of Radiology both of Tianjin First Central Hospital and YouAn hospital affiliated of Capital medical university. The research protocol was approved by the Hospital Research Ethics Committees.

All of the patients were diagnosed with AIDS based on positive HIV-1 antibody. The diagnosis of cryptococcal meningoencepgalitis was based on at least one of the following 3 findings: (1) isolated *C. neoformans* after cerebrospinal fluid (CSF) culture; (2) positive CSF cryptococcal antigen (Ag) titer; (3) positive CSF India ink staining and clinical symptoms of meningitis.^5^ All of the patients received plain and contrast MR imaging, with imaging data available. MR imaging was performed 2 weeks before or after the clinical diagnosis of cryptococcal meningoencephalitis. None of the patients had a history of receiving antifungal therapy. In 1 case with cerebellar lesion, operation had been done.

Based on the above criteria for enrollment, 69 patients were enrolled. However, due to no abnormalities detected by MR imaging in 14 patients, 55 patients were finally eligible as the subjects of our study, including 42 men and 13 women, with their ages of 40 ± 13 years ranging from 21 to 71 years. We also collected clinical data, such as the time of HIV diagnosis, therapeutic history of HAART, and CD4 T cell count.

MR imaging of the brain was performed on a 3.0 Tesla unit (Trio Tim, Siemens AG, Erlangen, Germany). The sequences included sagittal T1-weighted spin-echo, T2-weighted turbo spin echo, fluid attenuated inversion recovery (FLAIR), echo-planar diffusion-weighted imaging (DWI-EPI, *b* = 1000 s/mm^2^). Gadolinium-DTPA was injected via antecubital vein at a dose of 0.1 mmol/kg magnevist (Bayer, Germany) with a 20 mL saline flush. The injection rate was 2 mL/s by using a power injector.

### Images Analysis

All of the images were evaluated by 2 neuroradiologists with 5-year experiences in diagnosing infection diseases complicating AIDS. They were informed that all the patients had been definitively diagnosed with CNS cryptococcal infection. Agreement was reached by discussion for the imaging characterization.

Some of the studies reported that the MRI appearances of HIV patients with treatment of HAART were different from that of pre-HAART era.^17–19^ In the pre-HAART era, the lesion mainly manifested as dilated Virchow–Robin spaces and cryptococcomas and focused in the basal ganglia and brain stem with no contrast enhancement detected due to the immunodeficiency.^11,12,15,16^ Some studies have reported that HIV-infected patients with HAART show typical MRI signs of leptomeningitis or meningoencephalitis in the superficial part of brain.^17,19,20,27,28^ So in this large cohort study, we want to explore if different MRI distribution was correlated with the use of HAART and body immunity. Based on distribution of the lesions detected by MR imaging, the subjects were divided into 2 groups. Group1 includes patients with their lesions involving the central parts of brain, such as the basal ganglia, thalamus, hypothalamus, brain stem, or central cerebellum. The lesions were visualized to be dilated Virchow–Robin spaces, pseudocysts, or hazy brain base. Group 2 consisted of patients with lesions involving the superficial cerebral lobes and cerebellar hemisphere and the lesions have contrast enhancement. The distribution of the lesions was characterized based on FLAIR, DWI, and contrast imaging. The presence of atrophy was also denoted during image analysis.

### Definitions of Dilated Virchow–Robin Space, Pseudocyst, and Hazy Brain Base

**FIGURE 1:**
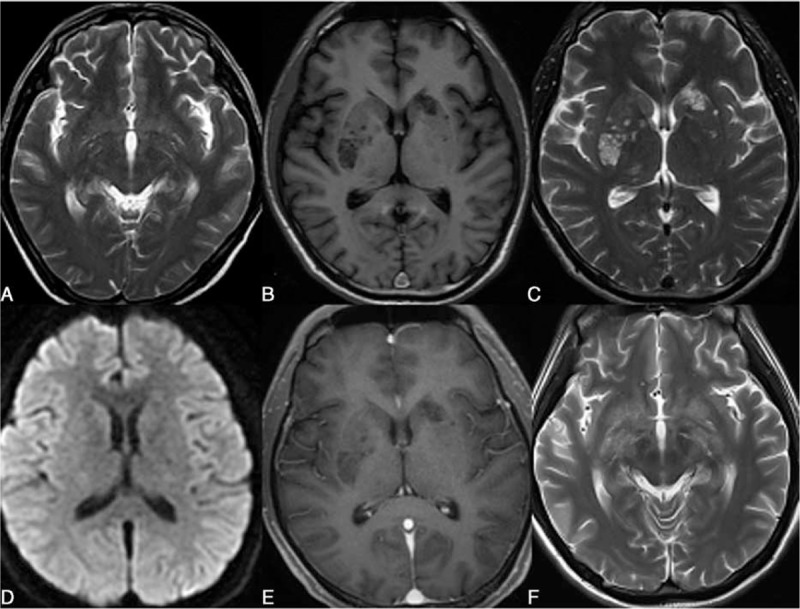
(A) Typical dilated Virchow–Robin spaces in HIV-1-infected patients with cryptococcal meningoencephalitis. Multiple dot-like lesions are demonstrated at the bilateral basal ganglia. The lesions show hyperitensity on T2WI (A). (B) Typical peudocysts in HIV-infected patients with cryptococcal meningoencephalitis. Multiple round lesions are seen in the bilateral basal ganglia. The lesions are demonstrated with low intensity on T1WI (B), hyperintensity on T2WI (C), no limited diffusion (D), and no contrast enhancement (E). (F) Typical hazy brain base in HIV-infected patients with cryptococcal meningoencephalitis. Symmetrical patchy lesions with poorly defined boundaries are seen in the bilateral basal ganglia. The lesions manifest hyperintensity on T2WI (F). The lesions are seen with growth along the perivascular spaces.

The well-circumscribed, round to oval shaped low-density lesion with a size of <2 to 3 mm in diameter (on the axial T2-weighted scan) and no limited diffusion were defined as dilated Virchow–Robin space (VRS) (Figure 1A). Pseudocyst (Figure 1B–E) was defined as such lesion with a diameter of >3 mm. Hazy brain base was defined by diffuse hyperintensity on T2WI and FLAIR involving parenchyma of basal brain (basal ganglia, thalamus, hypothalamus, and midbrain) (Figure 1F).^19^

### Definition of Leptomeningitis or Meningoencephalitis

**FIGURE 2:**
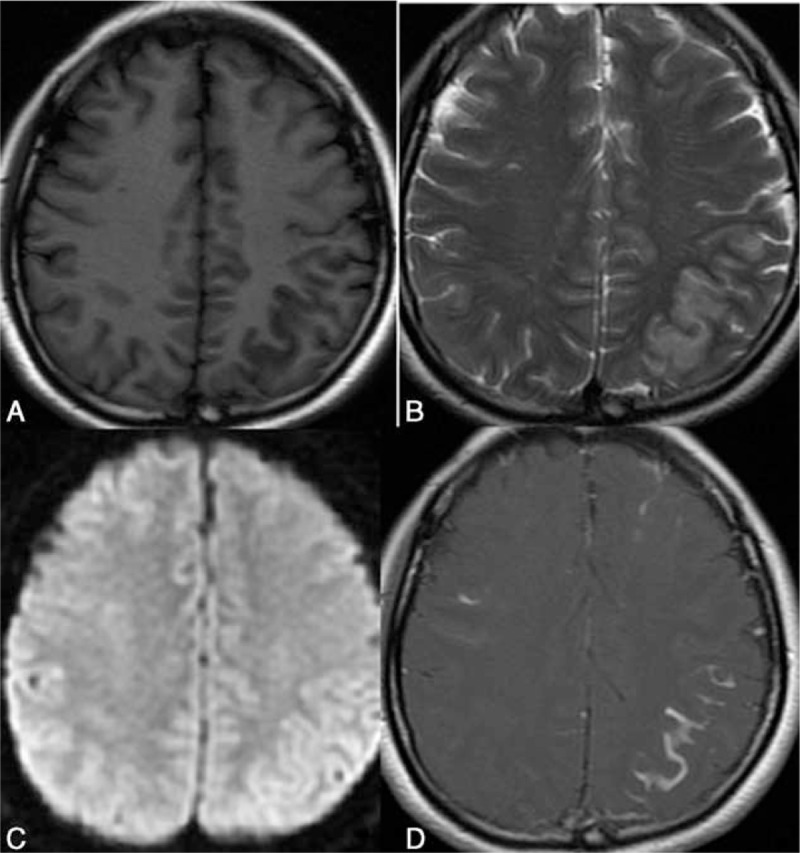
A 42-year-old woman with complaints of headache, fever, and vomiting. Both HIV antibody and cryptococcus were detected and found to be positive after culture of CSF. Left parietal and frontal cortex is shown with swelling; and the adjacent sulcus is shown to be narrowed. The lesions manifest low intensity on T1WI (A), hyperintensity on T2WI (B), no limited diffusion (C), and linear contrast enhancement (D). Abnormal contrast enhancement of the right frontal lobe is also demonstrated.

T2WI and FLAIR hyperintensity within the superficial brain lobes or cerebellum with regional meningeal enhancement was recognized as lesion of leptomeningitis or meningoencephalitis (Figure 2).

### Statistical Analysis

Two sample *t* tests were used to compare the ages of the 2 groups. Fisher's exact test was used to compare gender, history of HAART treatment, the presence of atrophy, and Virchow–Robin Space between the 2 groups. The Wilcoxon rank sum test was used to compare the CD4 T cell count and the time period since HIV detection between the 2 groups. A *P* value <0.05 was considered to be statistically significant.

## RESULTS

A total of 34 patients were included in Group 1 with the ages of 39 ± 15 years. This group was composed of 27 men and 7 women. In the other group, Group 2, there were 21 patients with the ages of 42 ± 12 years including 16 men and 5 women. No statistical difference was detected in terms of age and gender between the 2 groups.

The CD4 T cell count was 11.67 cells/mm^3^ (3.00–52.00 cells/mm^3^) and 42.00 cells/mm^3^ (10.00–252.00 cells/mm^3^) in Groups 1 and 2, respectively. Statistical difference of CD4 T cell counts was found between the 2 groups (*P* = 0.023). In Group 1, 13 patients had a therapeutic history of HAART, whereas 12 in Group 2. Group 2 patients received HAART therapy more frequently than Group 1 (*P* = 0.021). Virchow–Robin Space and atrophy were equally detected in both groups, with no statistical difference (Table 1).

**TABLE 1:**
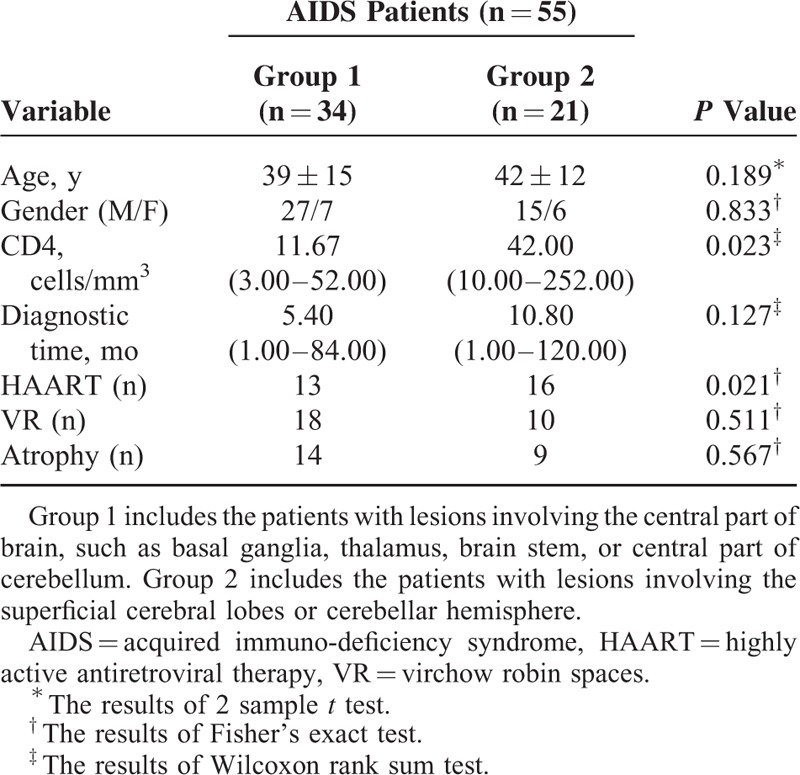
Demographical and Clinical Data of Enrolled Patients

Among the 34 patients in Group 1, 26.5% (9/34) was detected with Virchow–Robin spaces that only involved the basal ganglia. In these 9 patients, 55.6% (5/9) showed symmetrical lesions. In other 55.9% (19/34) of the patients, the abnormality of pseudocysts was shown, and the cerebellum was involved in 1 case. In these 19 patients, 42.1% (8/19) showed symmetrical lesions. For the remaining17.6% (6/34) of the patients, signs of hazy brain base were detected and all the lesions were found symmetrical. In all patients of Group 1, the lesions were shown with no or minimal contrast enhancement (Figure 1).

**FIGURE 3:**
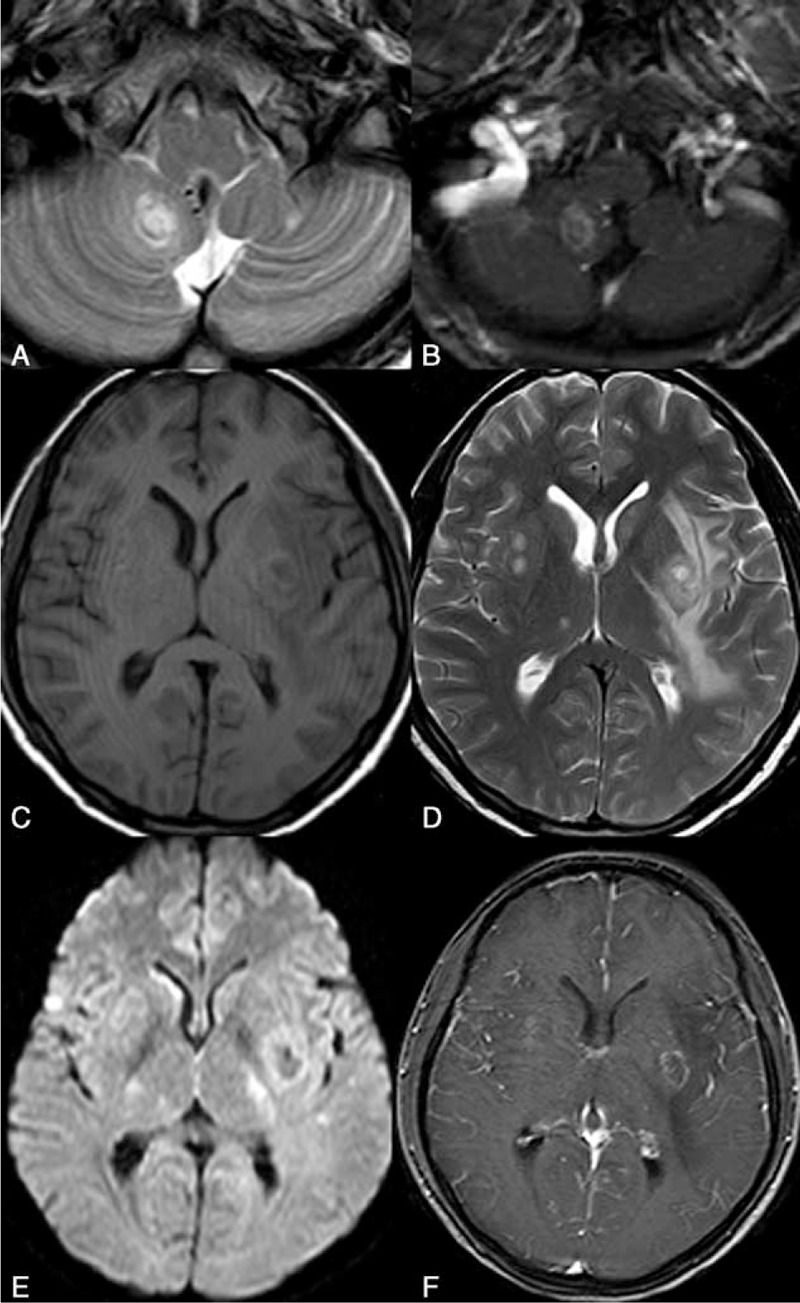
A 25-year-old woman with complaints of headache, fever, and vomiting. Both HIV antibody and cryptococcus were detected and found to be positive in the CSF. Multiple nodular lesions are shown in the right cerebellum and bilateral basal ganglia on MRI. The lesions manifests low intensity on T1WI (C), hyperintensity on T2WI (A, D), no limited diffusion (E), and circular contrast enhancement (D).

All the patients of Group 2 showed obvious meningeal contrast enhancement and parenchymal enhancement. The lesions were found with common involvements of left frontal lobe (12/21), right frontal lobe (11/21), parietal lobe (10/21), and cerebellum (12/21). In 23.8% (5/21) of the patients, multiple brain abscesses were simultaneously detected (Figure 3) (Table 2). In 1 special case, local cranial bone destruction, diffuse dural thickening, and abnormal contrast enhancement were demonstrated. Pathology in another case with cerebellar lesion showed chronic granulomatous response containing large quantity of lymphocytes, macrophages, and foreign organisms.

**TABLE 2:**
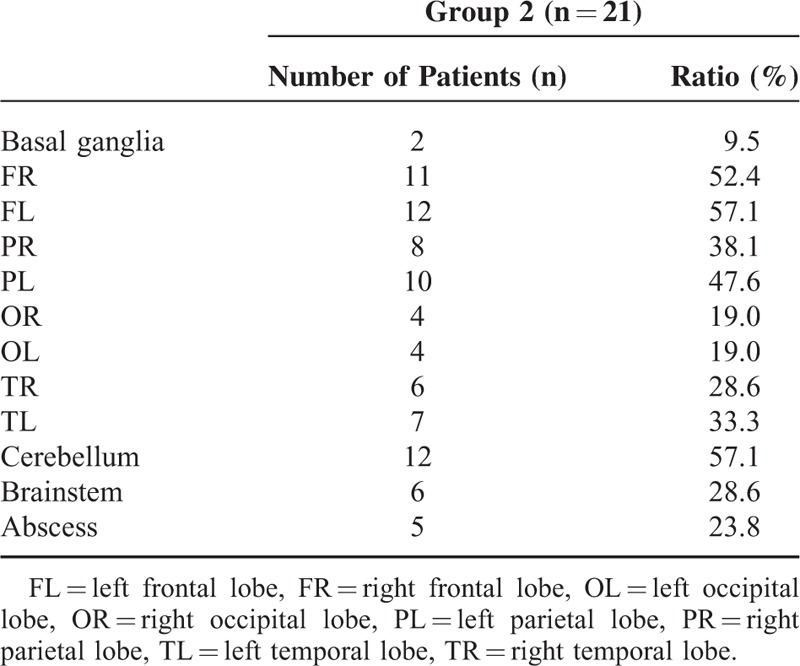
Distribution of Lesions in Superficial Cerebral Lobes and Cerebellum

## DISCUSSION

In the present study, the MRI imaging characterization from a large cohort of HIV-infected patients with crytococcal meningoencephalitis was analyzed. Based on distribution of the lesions, the patients were divided into 2 groups. The patients with superficial meningoencephalitis had significantly higher CD4 T cell count and more frequently use of HAART than the patients with central brain involvement.

As is known, the patients with AIDS sustain compromised cell-mediated immunity and are vulnerable to CNS cryptococcal infection. Uicker et al^22^ reported that CD4 T cell count is an important factor indicating acquired immune response against *C. neoformans* in an animal model of cryptococcal meningoencephalitis. CD4 T cell contributes not only for clearance of cryptococci but also for leukocytic infiltration into the lesion of its CNS infection. In our present study, the patients with higher CD4 T cell count were shown with more obvious leptomeningitis or meningoencephalitis, indicating a good response to infection of *C. neoformans*. It has also been reported that no or mild meningeal contrast enhancement is demonstrated in HIV-infected patients due to their immunodeficiency and immunogenic incompetence in recognizing polysaccharide capsule of cryptococcal organism.^11,12,24^ In our group of patients with lower CD4 T cell count, the lesions commonly involved the central part of brain, with no detected contrast enhancement that is related to the compromised response to the infection.

Since the use of HAART in the year of 1997, the incidence of cryptococcal infection in CNS of AIDS patients has dramatically decreased across the world. The imaging appearances of cryptococcal infection in CNS are totally different from the pre-HAART era. The reason may be related with recovered host immunity triggering inflammatory responses to cryptococcal antigens.^25,26^ High levels of inflammatory cells in CSF are found which also correlates with meningeal enhancement.^27^ Some studies have reported that HIV-infected patients undergoing treatment show typical MRI signs of leptomeningitis or meningoencephalitis.^17,19,20,27,28^ Another study reported that treatment by HAART has no impact on the lesions demonstrated by imaging.^18^ However, in our present study, the patients undergoing a history of HAART are more vulnerable to combined with leptomeningitis or meningoencephalitis. Our findings are different from that previously reported possibly due to CD4 count of the patients and duration of HAART use.

In the pre-HAART era, Virchow–Robin space, pseudocyst, and hazy brain base are the most common lesions in HIV-infected patients with CNS crytococcal infection.^13,15,16,29^ Such lesions are commonly located at the basal ganglia and brain stem. The cerebellum can also be involved. Budding yeast such as cells and mucoid substances are found filling in the Virchow–Robin spaces whose enlargements are able to result in pseudocysts. Soap-bubbles such as sign is detectable when multiple cysts accumulate together.^14^ The lesion of hazy brain base was first described by Katchanov as parenchymal edema caused by fungal involvement to basal ganglia along the perivascular spaces.^19^They also reported that the sign is often concurrent with infarction due to vasculitis of the minor perforating arteries. However in our subjects, 6 were demonstrated with hazy brain base, and its concurrence with infarction was found in only 2 cases.

In the group with superficial distribution of the lesions, frontal lobe, parietal lobe, and cerebellum were the most commonly involved, especially cerebellum, with an involvement rate of 57.1%. Abscess had not been reported in the previous literature and concurrent abscess was found in our 5 cases, of which 1 case showed chronic granulomatous response containing large quantity of lymphocytes, macrophages, and foreign organisms. The presence of abscess also indicates the recovery of immunity after use of HAART and robust immune response to the infection.

Additionally, the present study does have some limitations. First, no follow-up data were collected to show the natural evolution and prognosis of the lesions in different groups of patients. Second, the clinical data, such as CSF culture, the duration of HAART use, and the occurrence of complications in other systems should be further investigated in correlation to MRI findings. Third, concurrence of IRIS with cryptococcal meningoencephalitis after the use of HAART could be further investigated for their differentiation. Fourth, further normal findings on MRI in HIV patients with cryptococcal meningoencephalitis should also be collected to compare the difference of CD4 counts and use of HAART.

## CONCLUSION

In conclusion, central and superficial distributions of the lesions detected by MR imaging in HIV-1-infected patients with cryptococcal meningoencephalitis are correlated with the CD4 count and the use of HAART. The basal ganglia are the most commonly involved location in the group with centrally distribution. The frontal, parietal lobe, and cerebellum are the most commonly involved locations in the group with superficially distribution.
